# Anticancer Gold(III) Compounds With Porphyrin or N-heterocyclic Carbene Ligands

**DOI:** 10.3389/fchem.2020.587207

**Published:** 2020-11-06

**Authors:** Ka-Chung Tong, Di Hu, Pui-Ki Wan, Chun-Nam Lok, Chi-Ming Che

**Affiliations:** ^1^State Key Laboratory of Synthetic Chemistry, Department of Chemistry, The University of Hong Kong, Hong Kong, China; ^2^Laboratory for Synthetic Chemistry and Chemical Biology, Health@InnoHK, Hong Kong, China

**Keywords:** gold(III), porphyrin, N-heterocyclic carbene, anticancer, biomolecular target, formulation, biosensing

## Abstract

The use of gold in medicine has a long history. Recent clinical applications include anti-inflammatory agents for the treatment of rheumatoid arthritis (chrysotherapy), and is currently being developed as potential anticancer chemotherapeutics. Gold(III), being isoelectronic to platinum(II) as in cisplatin, is of great interest but it is inherently unstable and redox-reactive under physiological conditions. Coordination ligands containing C and/or N donor atom(s) such as porphyrin, pincer-type cyclometalated and/or N-heterocyclic carbene (NHC) can be employed to stabilize gold(III) ion for the preparation of anticancer active compounds. In this review, we described our recent work on the anticancer properties of gold(III) compounds and the identification of molecular targets involved in the mechanisms of action. We also summarized the chemical formulation strategies that have been adopted for the delivery of cytotoxic gold compounds, and for ameliorating the *in vivo* toxicity.

## Introduction

The medicinal use of gold against disease has been recorded since ancient times. In the early twenty century, the discovery of the antiarthritic activity of gold(I) complex (sodium gold(I) thiopropanol-sulfonate; Allochrysine) led to the development of clinically useful gold(I)-thiolate drugs including sodium aurothiomalate (Myochrysine) and the acetylated glucose derivative of the gold(I)-phosphine complex (Auranofin) for the treatment of rheumatoid arthritis. Since the serendipitous discovery of the therapeutic value of cisplatin, cisplatin-based chemotherapy has been widely used in treatment against various types of cancers (Kelland, 2007; Hill and Sadler, 2016). Extensive studies on mechanisms of action have demonstrated that cisplatin covalently interacts with DNA, activates DNA damage response, inhibits DNA repair mechanisms, and consequently results in cellular apoptosis (Jung and Lippard, 2007). However, the issues of dose-limiting toxicity and chemoresistance to cisplatin remain challenging in clinical practice (Rabik and Dolan, 2007). Recently, there has been an upsurge of interest in the development of gold compounds for anticancer applications due to the cytotoxicity of the gold complex against cancer cells. Particularly, the isoelectronic nature (d^8^) of gold(III) with platinum(II) suggests that gold(III) compounds may share similar properties with platinum(II)-based anticancer agents. Nonetheless, the instability and reactivity of the gold(III) ion, such as a facile reduction into gold(I) or gold(0) *via* intracellular redox reactions under physiological conditions, hamper the therapeutic application of these gold(III) compounds. Over the past few decades, we have made use of strong electron-donor ligands, such as porphyrin, pincer cyclometalated (C^∧^N^∧^C), and N-heterocyclic carbene, for the preparation of cationic gold(III) compounds. All of them exhibit good stability in physiological environments and display a promising anticancer potency against a broad spectrum of cancer cells derived from human tumor tissues. More importantly, distinct from traditional platinum-based therapeutics targeting DNA *via* non-repairable interactions, the unique stable gold(III)–ligand coordination scaffold allows the complexes to bind to protein target(s) relevant to cancer cell survival and proliferation, and hence, leads to functional inhibition and associated anticancer activities.

## Anticancer Gold(III) Porphyrin Complexes

**Figure 1:**
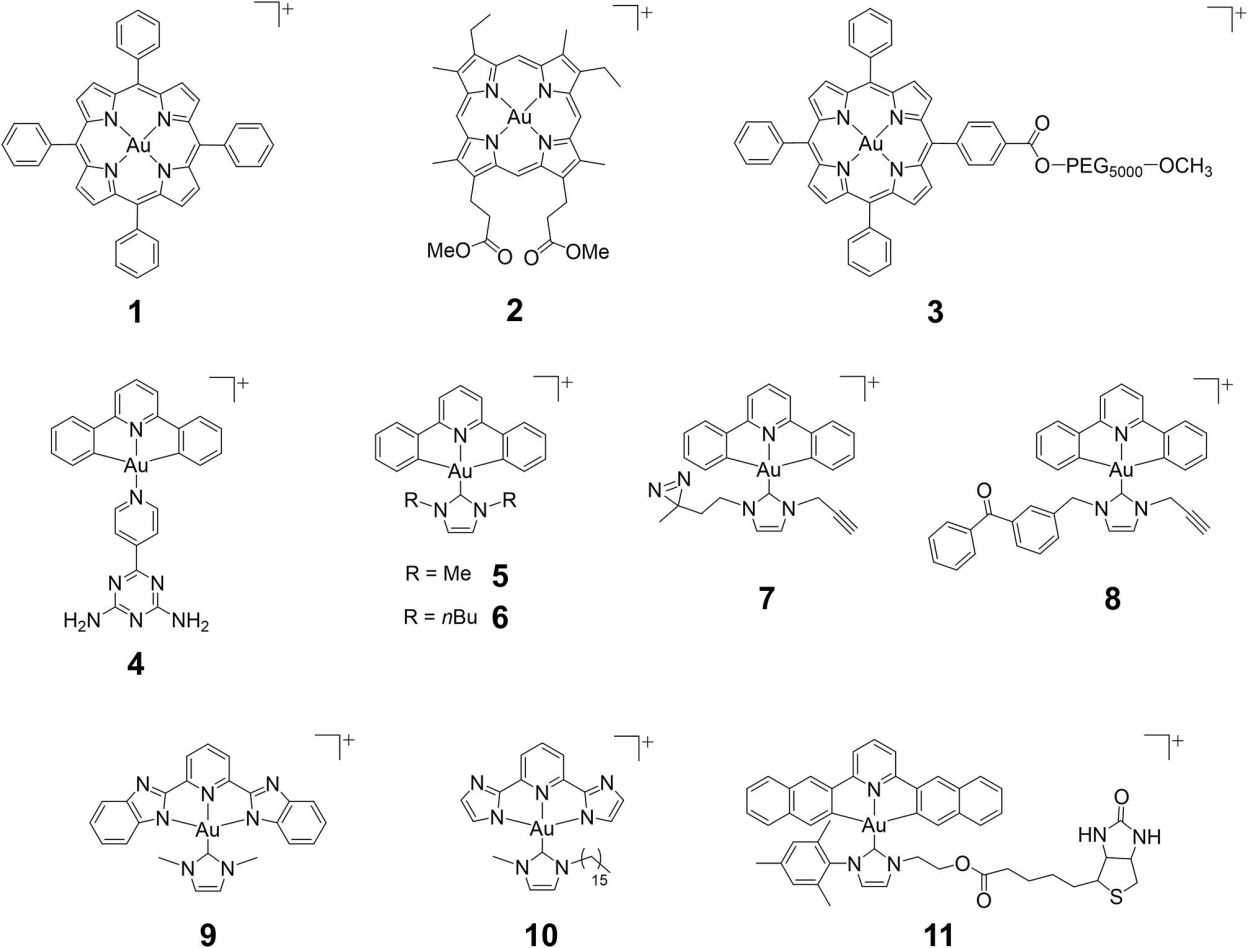
Chemical structures of cationic gold(III) complexes with porphyrin, pincer cyclometalated and N-heterocyclic carbene (NHC) ligands.

**Figure 2:**
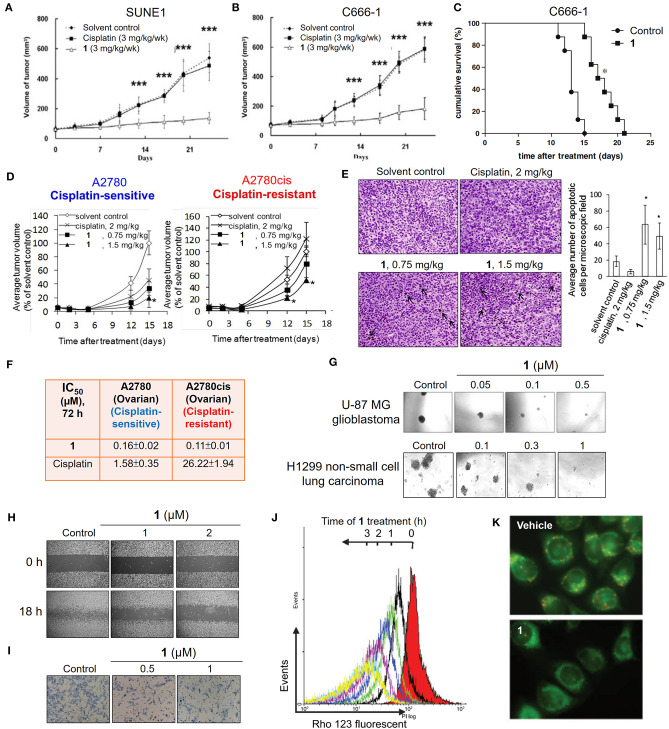
Anticancer properties of the cationic gold(III) porphyrin complex. Tumor volumes of mice bearing **(A)** nasopharyngeal SUNE1, **(B)** C666-1 or **(D)** ovarian (cisplatin-sensitive A2780 and–resistant A2780cis) xenografts after treatment with **1** or vehicle control. **(C)** Survival curves of mice bearing NPC C666-1 metastatic tumors in control and **1**-treated groups. **(E)** TUNEL staining of A2780cis tumor tissues of mice in different groups (left; arrows indicate the apoptotic cells). Bar chart illustrates the average number of apoptotic cells per microscopic field in different groups (right). **(F)** *In vitro* cytotoxicity (IC_50_, 72 h) of **1** and cisplatin on ovarian (cisplatin-sensitive and -resistant) cancer cell lines. **(G)** Representative images of the inhibitory activities of **1** in sphere formation by human glioblastoma U-87 MG and non-small cell lung cancer H1299 cells. **(H)** Antimigratory and **(I)** antimetastatic activities of **1** on the cell migration and invasion by NPC C666-1 cells. **(J)** Flow cytometric analysis of Δψ_m_ depolarization in **1**-treated HONE1 cells by fluorescent Rho-123 probe. **(K)** Fluorescence imaging of Δψ_m_ depolarization in **1**-treated cells by mitochondrial membrane potential JC-1 probe. **(A,B)** Reprinted with permission from To et al. (2009). Copyright 2010, Elsevier. **(C,H,I)** Reprinted with permission from Lum et al. (2010). Copyright 2010, Elsevier. **(D,E)** Reproduced from Lum et al. (2014) with permission from the Royal Society of Chemistry. **(G)** Reproduced from Lum et al. (2013) with permission from the Royal Society of Chemistry. **(J,K)** Adapted from Wang et al. (2005). Cancer Res, Vol. 65, Article ID CAN-05-2867, (2005). Data are presented as mean ± SEM (**A,B**; *n* = 12; Student's *t* test; ***p < 0.005, compared with vehicle control group), (**C**; *n* = 8; Student's *t* test; *p = 0.0001, compared with vehicle control group), (**D**; **1**, *n* = 3; vehicle control, *n* = 8; Student's *t* test; *p ≤ 0.05, compared with vehicle control group) and (**E**; **1** (0.75 mg/kg), *n* = 3; **1** (1.5 mg/kg), *n* = 6; vehicle control, *n* = 11; Student's *t* test; *p < 0.05, compared with vehicle control group).

In 2003, an anticancer gold(III) porphyrin system was established that was exemplified by a gold(III) *meso*-tetraphenylporphyrin complex [denoted **Au-1a** (**1**); Figure 1], demonstrating potential clinical applications (Che et al., 2003). The porphyrin ligand can stabilize the gold(III) ion against demetalation and reduction by the biological reductant glutathione (Sun et al., 2010). *In vitro* biological studies have proven the high anticancer potency of gold(III) porphyrin complexes against a wide range of cancer cell lines, such as neuroblastoma, melanoma, ovarian, breast, colorectal, lung, and nasopharyngeal cancers, with IC_50_ values at low micromolar or even nanomolar levels (Lammer et al., 2015; Toubia et al., 2019). Moreover, *in vivo* studies have demonstrated the promising tumor growth inhibitory ability of gold(III) porphyrins in different mouse models of cancer (Figures 2A,B,D) and the induction of apoptosis in tumor xenograft tissues (Figure 2E). Compared to the clinically used cisplatin, complex **1** displays higher cytotoxicity with IC_50_ values significantly lower than those of cisplatin and is equally active toward both cisplatin-sensitive and -resistant cancer cells (Figure 2F; Lum et al., 2014). Multiple mechanisms of chemoresistance to cisplatin involve a reduced intracellular accumulation, sequestration/detoxification by thiols (e.g., glutathione), increased DNA damage repair response, and efflux transports (Galluzzi et al., 2012). The lack of cross-resistance to cisplatin suggests that the stable gold(III) porphyrin complex penetrates into the cancer cells and exerts anticancer activities *via* different mechanisms from cisplatin. Furthermore, **1** is capable of blocking the self-renewal of cancer stem-like cells (Figure 2G; Lum et al., 2013), suppressing angiogenesis *in vitro* and *in vivo* (Lum et al., 2011), inhibiting cancer cell migration (Figure 2H), invasion and metastasis (Figure 2I), and prolonging the survival lifetime of nasopharyngeal carcinoma metastasis-bearing mice (Figure 2C; Lum et al., 2010).

**Figure 3:**
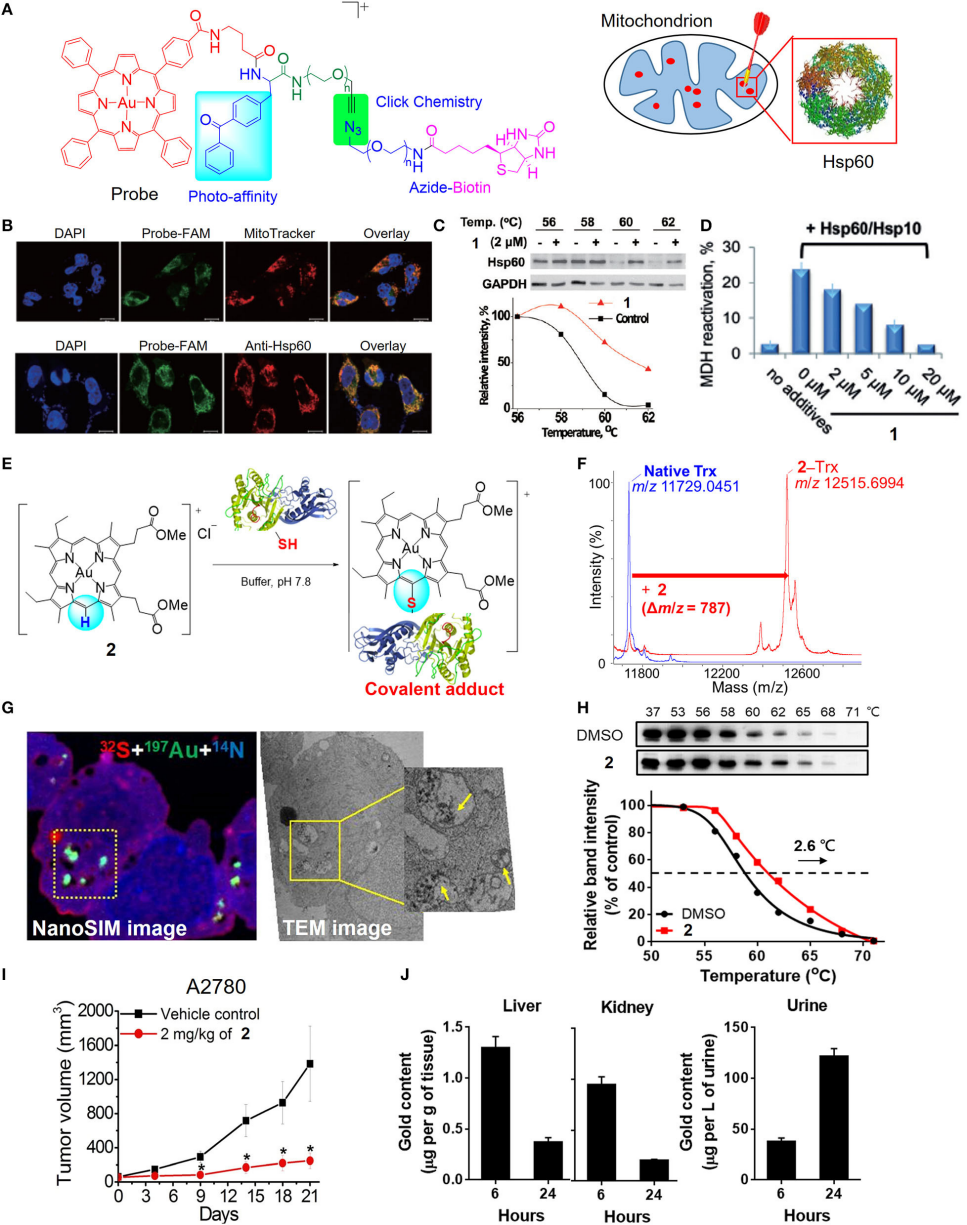
Target engagement of anticancer gold(III) porphyrin complexes. **(A)** A clickable photoaffinity probe for the target identification of **1**. **(B)** Fluorescence imaging for colocalization of the probe with MitoTracker Orange and fluorescence-labeled Hsp60. **(C)** Thermal stabilization of Hsp60 after treatment of HeLa cells with **1** as determined by CETSA. **(D)** Inhibition of Hsp60 chaperone activity by **1**. **(E)** Proposed reaction scheme of **2** with protein cysteine thiol under physiological conditions. **(F)** MALDI-TOF-MS spectra of thioredoxin (Trx) before and after reaction with **2**. **(G)** NanoSIMS and EM imaging of the sulfur-rich, electron-dense aggregates in degraded mitochondria of **2**-treated cells. **(H)** Thermal stabilization of PRDX3 following treatment of A2780 ovarian cancer cells with **2** as determined by CETSA. **(I)** *In vivo* antitumor activity of **2** and **(J)** biodistribution of gold content in mice after treatment with **2**. **(A–D)** Reprinted with permission from Hu et al. (2016). Copyright 2015, John Wiley and Sons. **(E–J)** Reprinted with permission from Tong et al. (2020). Copyright 2020, National Academy of Sciences. Data are presented as mean ± SEM (**I**; *n* = 5; Student's *t* test; *p < 0.05, compared with vehicle control group).

In previous studies, a number of approaches, including biochemical analyzes, transcriptomics, and proteomics, have been utilized to investigate the mechanisms of the anticancer action of gold(III) porphyrins. Several lines of evidence have revealed that complex **1** can target mitochondria. Treatment of cancer cells with **1** induces a rapid depletion of mitochondrial transmembrane potential (Figures 2J,K) and subsequently causes caspase-dependent and -independent apoptotic pathways (Wang et al., 2005). Cellular oxidative stress and shift of the balance between proapoptotic and antiapoptotic proteins were also observed in **1**-treated cancer cells. In addition, treatment of **1** can arrest the cell cycle progression in the G0/G1 phase, activate p38 mitogen-activated protein kinases (MAPK), and inhibit the redox regulation of thioredoxin reductase (TrxR) (Wang et al., 2007; Tu et al., 2009). To obtain deeper insights into the mechanisms of action of gold(III) porphyrins, efforts have been made to identify the direct molecular targets of **1** using a chemoproteomic approach with the aid of a clickable photoaffinity probe containing a benzophenone moiety (Figure 3A; Hu et al., 2016). In this regard, we identified a mitochondrial chaperone, heat shock protein 60 (Hsp60) as one of the molecular targets of **1** *in vitro* and *in cellulo*. The proposed non-covalent biomolecular interaction of **1** with Hsp60 was further supported by different binding studies including a cellular thermal shift assay, saturation-transfer difference NMR, and protein fluorescence quenching (Figures 3B,C). The dose-dependent inhibitory effect of **1** on the chaperone activity of Hsp60 in the reactivation of the denatured substrate of malate dehydrogenase (MDH) was also confirmed (Figure 3D). In addition, structure-activity relationship studies from the analogous gold(III) and platinum(II) complexes showed that gold(III) ion, porphyrin ligand, and the monocationic charge character governed by the central gold(III) ion play important roles for the inhibition of the chaperone activity of Hsp60. Our study provided a deeper understanding on the molecular targets of **1** in cancer cells, enabling the improvement of the anticancer activity of gold(III) porphyrins *via* structural modification.

By varying the *meso*-tetraphenylporphyrin ligand into quasiphysiological mesoporphyrin IX, we have uncovered a previously unknown biomolecular interaction of the gold(III) complex that can be exploited for anticancer applications (Tong et al., 2020). Gold complexes usually interact with cysteine thiols *via* M–S bond formation. Nonetheless, the gold(III) mesoporphyrin IX dimethyl ester (**2**) is unique in that the periphery (*meso*-carbon atom) of the porphyrin ligand is activated by the electrophilic gold(III) ion to undergo nucleophilic aromatic substitution with selectivity to the cysteine thiols of proteins, such as thioredoxin, relevant to cancer (Figures 3E,F). Notably, **2**-treated cancer cells resulted in the formation of gold-bound sulfur-rich protein aggregates in the cytosolic region and more specifically in mitochondria, as revealed by nanoscale secondary ion mass spectrometry (nanoSIMS) and electron microscopic imaging techniques (Figure 3G). Based on thermal proteome profiling mass spectrometry analysis and a cellular thermal shift assay (CETSA), potential protein targets including peroxiredoxin III (PRDX3) and deubiquitinase (UCHL3) were identified to engage with complex **2**, as supported by their increased thermal stability upon the treatment of **2** compared with vehicle control (Figure 3H). A series of biochemical experiments further validated the biological consequences of the treatment with **2** resulting in the inhibition of the protein activities, oxidative stress-mediated cytotoxicity, and the accumulation of ubiquitinated proteins. Importantly, **2** exhibited effective antitumor activities in two independent mouse models (Figure 3I) and demonstrated a favorable metabolism, biodistribution, and clearance conferred by the quasiphysiological mesoporphyrin IX ligand (Figure 3J). Taken together, these results demonstrated a new modality of cysteine targeting by the anticancer gold(III) complex.

## Formulations of Gold(III) Complexes for Antitumor Treatment

**Figure 4:**
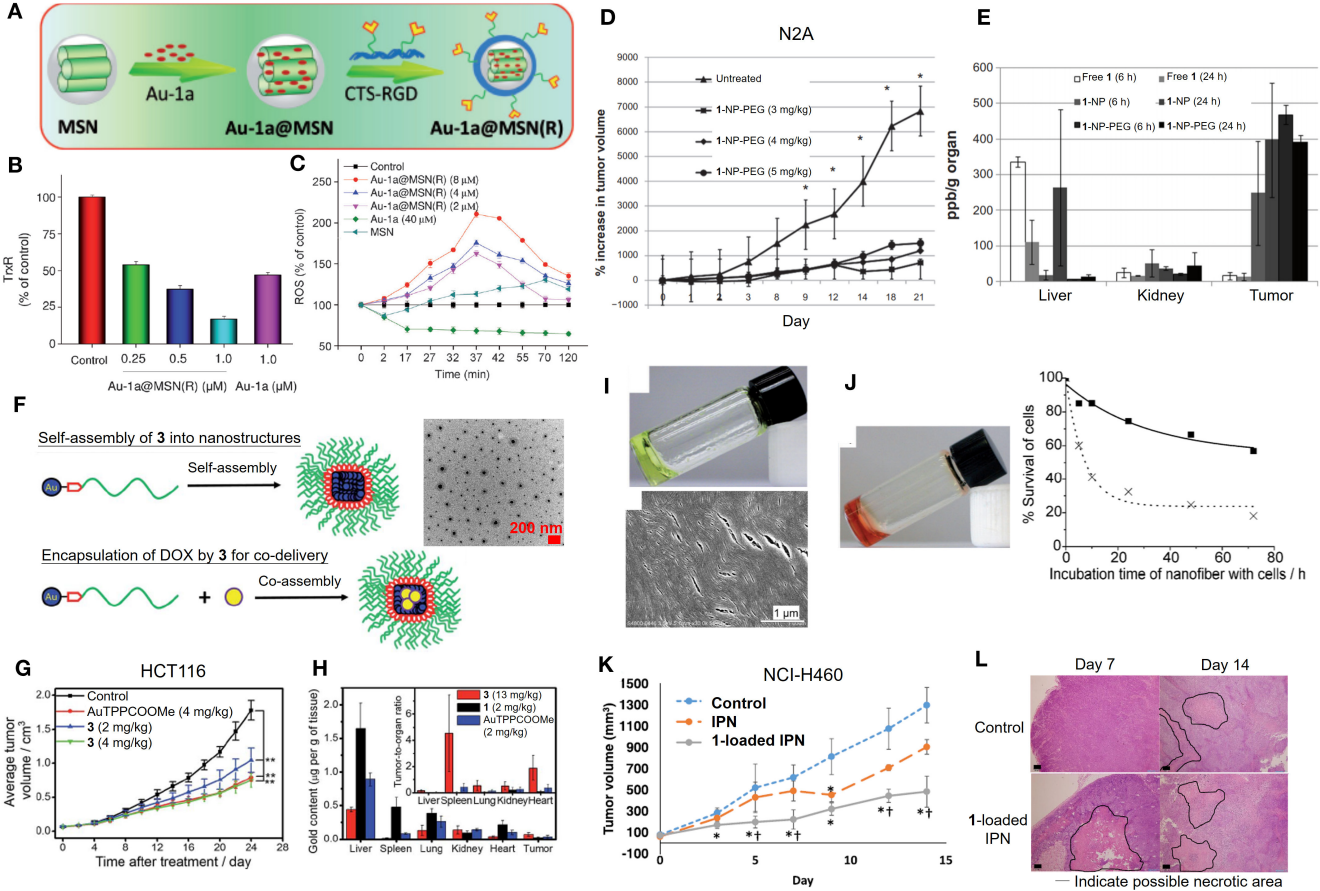
Formulations of anticancer gold(III) porphyrin complexes. **(A)** Preparation of **1**-loaded RGD peptide-grafted mesoporous silica nanoparticles, **Au-1a**@MSN(R). Dose-dependent **(B)** inhibitory effect of **Au-1a**@MSN(R) on the *in vitro* TrxR activity and **(C)** induction of intracellular ROS level. **(D)** Antitumor activity of **1**-encapsulated PEG-modified lipid nanoparticles (**1**-NP-PEG) and **(E)** biodistribution of gold content on N2A tumor-bearing mice. **(F)** Schematic diagram of self-assembly of **3** into nanostructures and as a carrier with the co-assembly of doxorubicin (yellow circle). **(G)** Antitumor activity of conjugate **3** in mice bearing HCT116 xenografts. **(H)** Biodistribution and tumor-to-organ ratio of conjugate **3** in tumor-bearing mice. **(I)** Photograph (top) and SEM image (bottom) showing formation of supramolecular polymer (**4-SP**) after cooling the heated solution (323 K) to 298 K. **(J)** Photograph of **Au-1a**-encapsulated supramolecular polymer **(4-SP-Au-1a)** after cooling the heated solution to 298 K (left). *In vitro* cytotoxicity of **4-SP** (solid line) and **4-SP-Au-1a** (dotted line) against melanoma B16 cancer cells in a time-dependent manner (0-72 h) (right). **(K)** Antitumor activity of **1**-loaded IPN in NCI-H460 xenografted mice. **(L)** Hematoxylin and Eosin (H&E) stain of tumor tissues from mice at day 14 post-treatment. **(A–C)** Reprinted with permission from He et al. (2014). Copyright 2014, John Wiley and Sons. **(D,E)** Adapted from Lee et al. (2012). Int J Nanomedicine, Vol. 7, Article ID S28783, (2012); licensed under a Creative Commons Attribution (CC BY-NC-ND) license. **(F–H)** Reproduced from Chung et al. (2017) with permission from the Royal Society of Chemistry. **(I,J)** Reprinted with permission from Zhang et al. (2012). Copyright 2012, John Wiley and Sons. **(K,L)** Reprinted with permission from Lee et al. (2019). Copyright 2019, Springer Nature. Data are presented as mean ± SEM (**D**; *n* = 5–6; Student's *t* test; *p < 0.05, compared with untreated group), (**G**; *n* = 8; Student's *t* test; **p < 0.01, compared with solvent control group), and (**K**; *n* = 6; Student's *t* test; *p < 0.05, compared with control group receiving UV exposure only; ^†^*p* < 0.05, compared with IPN group).

Although gold(III) porphyrin complexes were demonstrated to exhibit promising anticancer activities in various human cancer cell lines, further applicability on cancer therapy remains challenging due to its high toxicity in normal cells and tissues. Formulation of the gold(III) porphyrin complexes by nanotechnology-based delivery systems is a potential strategy to mitigate the challenges of their short half-life in blood circulation and rapid distribution into major organs (Figure 4). By making use of biocompatible materials, the encapsulation of gold(III) porphyrin in gelatin-acacia microcapsules improved the aqueous solubility and stability, as well as the *in vivo* antitumor efficacy of the compound. With the use of RGD tripeptide acting as a targeting ligand, we have also described a cancer-targeted mesoporous silica nanoparticle (MSN) as a delivery carrier for complex **1** (Figure 4A; He et al., 2014). The **1**-encapsulated MSNs coated with RGD-grafted polymeric chitosan [**Au**-**1a**@MSN(R)] displayed increased biocompatibility and colloidal stability in physiological media, and an enhanced cell-killing selectivity between cancer and normal cells. More importantly, **Au**-**1a**@MSN(R) exhibited an augmented inhibitory activity on TrxR (Figure 4B), elevated cellular oxidative stress (Figure 4C), and enhanced apoptosis-inducing efficacy. Poly(ethylene) glycol (PEG) is the most commonly employed hydrophilic and biocompatible polymer in the research of drug delivery systems (Knop et al., 2010). The use of PEG in a formulation can prolong the blood circulation half-life and provide a shield to the parental drug, avoiding rapid uptake by the organs (liver and spleen) of the reticuloendothelial system (RES) or clearance from the body (Harris and Chess, 2003). The advantages of which render PEG as an attractive material in the development of the formulation of improving the biodistribution and/or systemic toxicity of cytotoxic metal complexes. In this regard, we have reported the use of PEG surface-modified lipid nanoparticles made of the Brij 78 surfactant and cetyl alcohol for delivery of complex **1** into tumor xenografts of neuroblastoma (N2A) (Figure 4D; Lee et al., 2012). The PEGylated lipid nanoparticles (**1**-NP-PEG) enhanced the preferential tumor accumulation of complex **1** rather than in major organs (Figure 4E) and thereby, resulted in a higher tumor-killing efficacy when compared with free gold porphyrin treatment. Recently, we have also described a multifunctional PEGylated gold(III) porphyrin conjugate [Au(TPP-COO-PEG_5000_-OCH_3_)]Cl (**3**) that can self-assemble into nanostructures in aqueous media (Figure 4F; Chung et al., 2017). The cleavable ester linkage allows for the release of an active gold(III) porphyrin moiety from the conjugate *in cellulo*. This PEGylated conjugate displays a higher selectivity on different cancer cell lines over non-tumorigenic cells. Importantly, *in vivo* experiments have shown that conjugate **3** can significantly inhibit the growth of human colon cancer HCT116 xenografts (Figure 4G) or cisplatin-resistant ovarian cancer A2780cis xenografts in nude mouse models. The enhanced permeability and retention effect, which is a pathophysiological characteristic of some solid tumors, presumably promotes the accumulation of the conjugate **3** in tumors rather than in normal organs (Figure 4H), leading to low systemic toxicity. Additionally, the self-assembled PEGylated gold(III) porphyrin conjugate can act as a nanocarrier for the co-delivery of chemotherapeutics such as doxorubicin, to achieve a strong synergistic anticancer activity. By harnessing the non-covalent intermolecular interactions (hydrogen-bonding and π-π interactions), a pincer gold(III) complex containing a hydrogen-bonding motif [Au^III^(C^∧^N^∧^C)(4-dpt)]^+^ (4-dpt = 2,4-diamino-6-(4-pyridyl)-1,3,5-triazine) (**4**) has been demonstrated self-assembling into a supramolecular polymer (**4-SP**) (Figure 4I), displaying sustained cytotoxicity with selectivity toward cancerous cells (Zhang et al., 2012). Intriguingly, such superstructures can also encapsulate cytotoxic agents such as complex **1** (**4-SP-Au-1a**) to achieve a sustained-release behavior for anticancer treatment (Figure 4J). Furthermore, recently, we have described a hydrogel formulation which forms an interpenetrating network system (IPN) *via* photoinitiated free radical polymerization for the delivery of gold(III) porphyrin **1** (Lee et al., 2019). Compared to free gold porphyrin, **1**-loaded IPN displayed a more effective cell-killing ability, inhibition of tumor growth, and suppression of angiogenesis in mice bearing lung cancer xenografts (Figures 4K,L), which are attributable to the controlled release property of the cross-linked hydrogel.

## Anticancer Cyclometalated Gold(III) N-Heterocyclic Carbene Complexes

**Figure 5:**
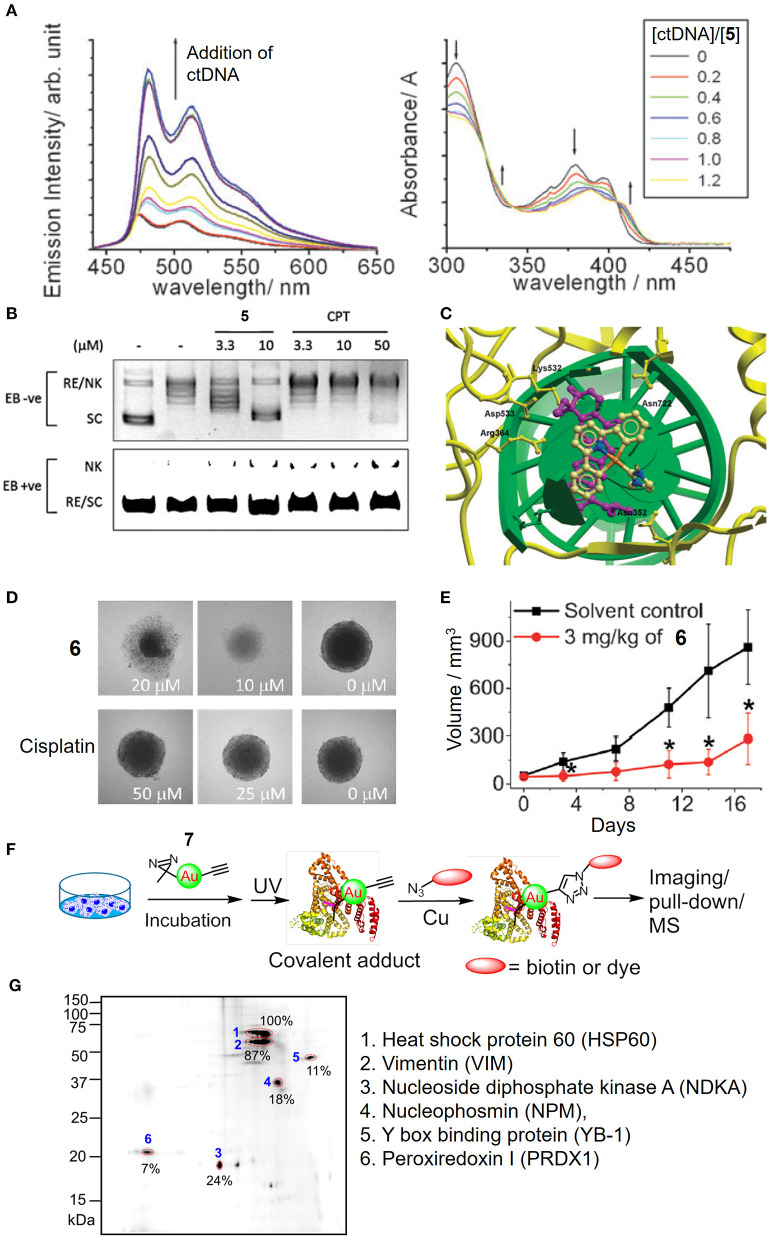
Anticancer properties of cyclometalated gold(III)–NHC complexes. **(A)** Emission (left) and absorption (right) titrations of complex **5** to ctDNA. **(B)** Dose-dependent inhibitory effect of **5** and camptothecin (CPT) on the TopoI-mediated DNA relaxation and induction of nicked DNA (EB, ethidium bromide; RE, relaxed; NK, nicked; SC, supercoiled). **(C)** Molecular modeling of **5** interacting with TopI-DNA (TopoI, yellow ribbon; DNA, green helix). Topotecan (purple) was also superimposed in the docking pose of **5**. **(D)** Images of HeLa cell spheroids after treatment of **6** or cisplatin at different concentrations for 72 h. **(E)** Tumor volume of HeLa xenograft-bearing mice after treatment of **6** for 17 days. **(F)** Schematic procedure of the identification of cellular protein targets of the gold(III)–NHC complex using diazirine-based probe **7**. **(G)** Fluorescence image of 2D gel electrophoresis of protein lysates from **7**-treated HeLa cells. The protein spots were visualized with an azide-Cy5 reporter through click reaction. **(A–C)** Reproduced from Yan et al. (2010) with permission from the Royal Society of Chemistry. **(D–G)** Reprinted with permission from Fung et al. (2017). Copyright 2017, John Wiley and Sons. Data are presented as mean ± SEM (**E**; **6**, *n* = 3; solvent control, *n* = 4; Student's *t* test; *p < 0.05, compared with solvent control group).

In addition to the tetradentate porphyrin ligands, stabilization of the gold(III) ion can also be achieved by coordination with the tridentate (C^∧^N^∧^C, C^∧^N^∧^N or N^∧^C^∧^N) pincer ligand containing deprotonated C-donor atom(s) (C^−^) and/or neutral σ-donating N-heterocyclic carbene (NHC) ligand(s), affording gold(III) complexes with high physiological stability and promising anticancer properties (Bertrand et al., 2017; Carboni et al., 2018; Bauer et al., 2019; Fares et al., 2020; Guarra et al., 2020). In this context, we have described the antitumor-active [Au^III^(C^∧^N^∧^C)(NHC)]^+^ (H_2_C^∧^N^∧^C = 2,6-diphenylpyridine) complexes (**5** and **6**). Complex **5** with a NHC ligand bearing two *N*-methyl substituents exhibits potent cytotoxicity with IC_50_ values at a low micromolar level on different human cancer cell lines and high selectivity by 167-fold lower in IC_50_ values to non-small cell lung carcinoma NCI-H460 relative to normal lung fibroblast CCD-19Lu cells (Yan et al., 2010). Mechanistic studies revealed that **5** interacts with DNA through intercalation (Figure 5A) and inhibits the topoisomerase I (TopoI) action on DNA relaxation (Figures 5B,C). Moreover, **5** treatment could effectively suppress the *in vivo* tumor growth of hepatocellular carcinoma xenograft model with no signs of toxicity such as body weight loss for a 28-day treatment. By varying the *N*-alkyl substituents on NHC into *N*-butyl groups, complex **6** is able to disintegrate the 3D cancer cells (HeLa) spheroids (Figure 5D) and significantly inhibit the tumor growth of cervical and lung carcinomas in two independent mouse models (Figure 5E; Fung et al., 2017). By employing a photoaffinity labeling-based chemoproteomics strategy, multiple protein targets have been identified to associate with the anticancer actions of pincer gold(III)–NHC complexes. For the clickable probes (**7** and **8**), the subtle modification of *N*-alkyl substituents on the NHC ligand with the photoaffinity diazirine or benzophenone group did not adversely affect the anticancer activity. Those molecular probes equipped with a photoaffinity group allow the engaged biomolecular targets to be covalently cross-linked with the gold(III)–NHC complex *via* UV light activation while the alkyne moiety functions as a ligation handle with an azido reporter for pull-down (biotin-streptavidin) or a photoluminescent labeling purpose *via* a copper(I)-catalyzed click reaction (Figure 5F). The diazirine-based probe **7** can label six (biotinylated) proteins from the two-dimensional protein blot of HeLa cervical cancer cell lysates as revealed by a fluorescent cyanine (azido-Cy5) reporter (Figure 5G). Based on tandem mass spectrometric analysis using MALDI-TOF mass spectrometry, the six labeled proteins were identified to be mitochondrial heat shock protein 60 (Hsp60), nucleophosmin (NPM), nucleoside diphosphate kinase A (NDKA), vimentin (VIM), peroxiredoxin I (PRDX1), and nuclease-sensitive element binding protein (Y box binding protein, YB-1), in which all of them are plausible anticancer targets. In contrast, four of which were labeled for probe **8** contained a benzophenone moiety. Based on a series of *in vitro* and cell-based experiments for target validation with unmodified complex **6**, the protein engagement was in line with the downstream biological responses. On the structural basis of molecular binding as revealed by molecular docking and hybrid quantum mechanics/molecular mechanics (QM/MM) studies, π-π interactions involving the pincer [Au^III^(C^∧^N^∧^C)]^+^ moiety of **6** and aromatic amino acid residues (i.e., Phe, Trp and Tyr) of the proteins were shown. It is worth noting that the analogous complexes [Pt^II^(C^∧^N^∧^N)(NHC)]^+^, [Pt^II^(N^∧^C^∧^N)(NHC)]^+^, and [Pd^II^(C^∧^N^∧^N)(NHC)]^+^ possessing similar structural scaffolds compete with the gold(III)–NHC probe **7** for the protein bindings *in cellulo*, implying that the monocationic character and stable orthogonal structure of the pincer-type metal–NHC complexes are crucial parameters for anticancer activities.

## Luminescent Probes for Biosensing

**Figure 6:**
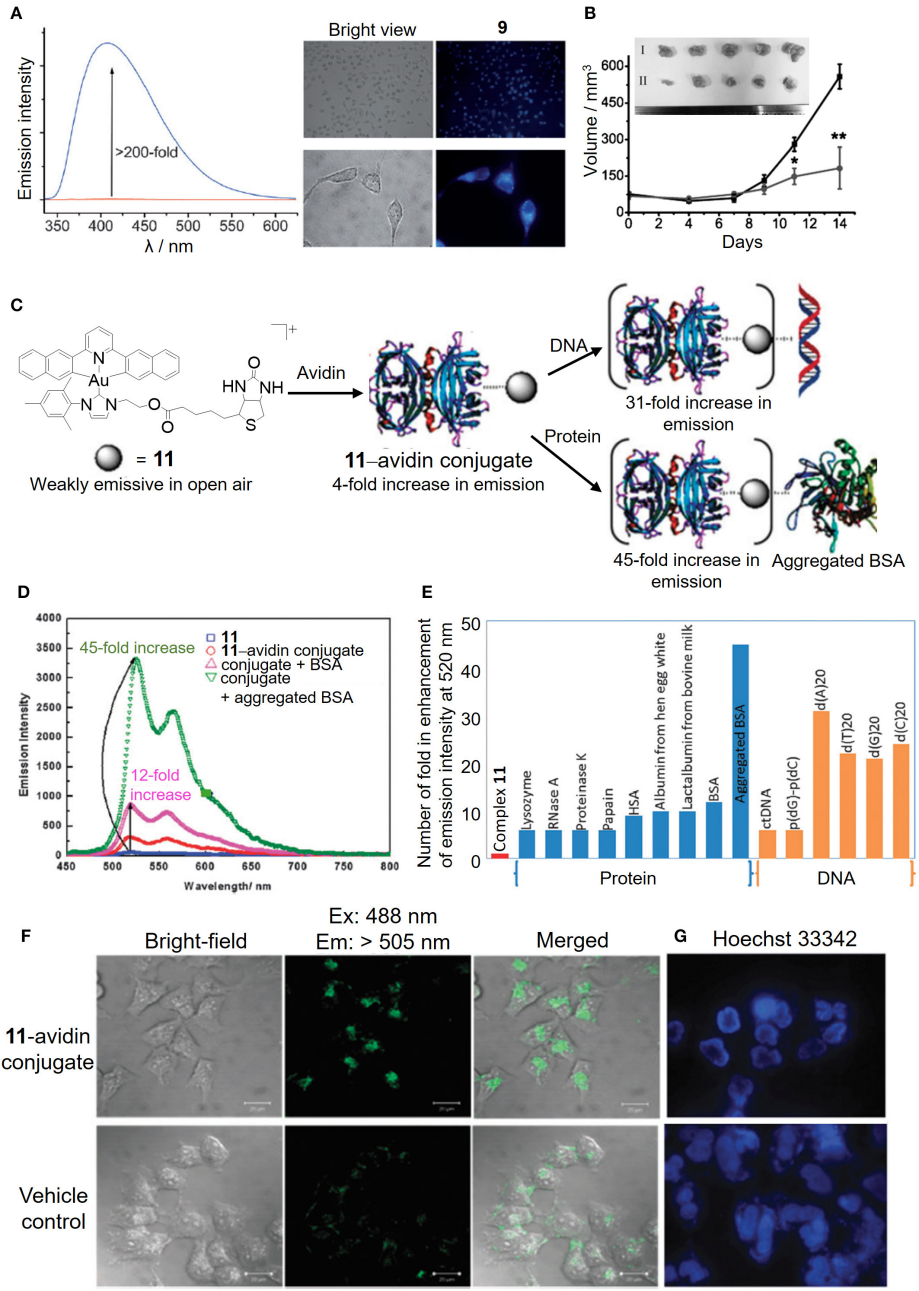
Luminescent properties of cyclometalated gold(III)–NHC complexes for biosensing. **(A)** Fluorescence intensity before (red line) and after (blue line) the addition of GSH to complex **9** in PBS (left). Fluorescence imaging of HeLa cells treated with vehicle control or complex **9** for 10 min (right). **(B)** Tumor volume of HeLa xenograft-bearing mice treated with vehicle control or complex **10** via intratumoral injection for 14 days. [Inset: Photos of tumors for vehicle control (I) and **10** (3 mg/kg) (II)]. **(C)** Schematic illustration of the bioconjugation of complex **11** bearing a biotin moiety to the avidin forming **11**–avidin conjugate for the biosensing of proteins or DNA. **(D)** Emission enhancement of the **11**–avidin conjugate after the addition of BSA or aggregated BSA. **(E)** Selective molecular sensing of the **11**–avidin conjugate with the single stranded DNA or aggregated BSA. **(F)** Fluorescence imaging of HeLa cells treated with the **11**–avidin conjugate. **(G)** Hoechst 33342 nuclear staining of the apoptotic HeLa cells after treatment with the **11**–avidin conjugate. **(A,B)** Reprinted with permission from Zou et al. (2013). Copyright 2013, John Wiley and Sons. **(C–G)** Reproduced from Tsai et al. (2015) with permission from the Royal Society of Chemistry. Data are presented as mean ± SEM (**B**; *n* = 5; Student's *t* test; *p < 0.05, **p < 0.01 compared with vehicle control group).

By judicious choice of the pincer ligands, as exemplified by the strongly fluorescent N^∧^N^∧^N (H_2_N^∧^N^∧^N: 2,6-bis(imidazol-2-yl)pyridine [H_2_IPI] and 2,6-bis(benzimidazol-2-yl)pyridine [H_2_BPB]) pincer ligands, we have developed a number of gold(III)–NHC complexes (**9** and **10**) with a switchable fluorescent property for the detection of cellular thiols (Zou et al., 2013). Owing to the low energy $5{\text{d}}_{{\text{x}}^{2}{-\text{y}}^{2}}$ orbital of the gold(III) ion, the gold(III)–NHC complexes are non-emissive in solution. In the presence of physiological thiols, the gold(III) was found to be reduced to gold(I) and accompanied by the release of the fluorescent pincer ligand, and hence gave strong emission enhancement even in live cells as monitored by fluorescence microscopy (Figure 6A). The gold(I) species can be stabilized by the coordinated NHC ligand(s) without further reduction and/or demetalation, and delivered to the biomolecular protein target (e.g., thioredoxin reductase) leading to effective *in vivo* antitumor activity (Figure 6B).

In addition to the fluorescent property derived from the pincer ligand, we have also taken advantage of the π-extended C-deprotonated C^∧^N^∧^C pincer and strong σ-donating NHC ligands in combination with the biotin–avidin interaction as a bioconjugation strategy to design a luminescent gold(III) NHC–avidin bioconjugate (Figure 6C; Tsai et al., 2015). The gold(III)–NHC complex (**11**) is weakly emissive in phosphate buffer with the emission maximum at 520 nm (Figure 6D). Functionalization of the NHC ligand with the biotin moiety renders the complex with high affinity for the bioconjugation of avidin. Protected by the protein scaffold, the luminescence quenching of the gold(III)–NHC complex by oxygen is attenuated and even displays significant emission enhancement with selectivity to single-stranded DNA and aggregated bovine serum albumin (Figure 6E). Moreover, such bioconjugation allows the luminescent gold(III)–NHC complex to be delivered by the protein carrier into the cytosolic region of cancer cells for anticancer activity (Figures 6F,G).

## Conclusions and Future Perspectives

Our works have demonstrated the use of porphyrin, pincer cyclometalated and/or N-heterocyclic carbene ligands for the stabilization of the electrophilic gold(III) ion and hence exhibit effective *in vitro* and *in vivo* antitumor activities. These unique lipophilic cations of gold(III) complexes with good stability under physiological conditions described herein display efficient cell penetration properties, a broad spectrum of anticancer activities in different human cancer cell lines, and effective *in vivo* antitumor responses in multiple mouse models. For target identification with the aid of the photoaffinity labeling-based chemoproteomics and cellular thermal shift proteomes, the anticancer gold(III) complexes consisting of the exposed gold(III) ion and a coordinated ligand scaffold are demonstrated to engage a number of anticancer molecular targets in association with expected downstream consequences of cancer suppression. Conceivably, these integrated approaches advance the understanding of the anticancer actions of gold(III) complexes, allowing for further structural optimization for targeted anticancer therapy. Taking advantage of the nanotechnology-based delivery system, the formulations of gold(III) complexes exhibit effective *in vivo* antitumor efficacies and improved biodistribution profiles, as well as ameliorate the systemic toxicity in mice caused by the free compounds. In addition, the resulting amphiphilic gold(III) complexes can self-assemble as drug carriers for the co-delivery of anticancer drugs to achieve synergistic therapy. It is envisaged that tumor targeting strategies through chemical modification can be adopted for the tumor-specific delivery of anticancer gold(III) complexes.

## Author Contributions

K-CT, DH, and P-KW wrote the article. C-NL and C-MC revised and edited the manuscript. All authors contributed to the article and approved the submitted version.

## Conflict of Interest

The authors declare that the research was conducted in the absence of any commercial or financial relationships that could be construed as a potential conflict of interest.
